# F248. COMMUNICATIVE-PRAGMATIC IMPAIRMENT IN SCHIZOPHRENIA: THE ROLE OF EXECUTIVE FUNCTION AND THEORY OF MIND

**DOI:** 10.1093/schbul/sby017.779

**Published:** 2018-04-01

**Authors:** Alberto Parola, Laura Berardinelli, Francesca M Bosco

**Affiliations:** 1 Center for Cognitive Science, University of Turin; 2 A.S.L. “Città di Torino”; 3 University of Turin, Institute of Neurosciences of Turin

## Abstract

**Background:**

Individuals with schizophrenia frequently exhibit a wide range of communicative-pragmatic disorders. Previous studies reported deicits in the comprehension of non-literal and figurative forms of language, such as indirect speech acts, deceit, irony, metaphors and idioms, as well as deficits in conversational and narrative skills. Moreover, schizophrenia is often associated with impairment in cognitive functions, such as Executive Functions (EF) and Theory of Mind (ToM). Few studies examined at the same time the role that ToM and EF can play in the comprehension of different communicative acts, such as sincere communicative acts, deceit and irony. Thus, the relation between ToM, EF and pragmatic ability in schizophrenia is still not completely clear. The aim of this study is to evaluate the relationship between the ability to manage different communicative pragmatic phenomena (i.e., sincere, deceitful and ironic communicative acts), and ToM and EF.

**Methods:**

26 individuals with schizophrenia and 26 matched controls took part in the study. We evaluated communicative pragmatic-ability using the lingusitic and extralinguistic scales of the Assement Battery for Communication (ABaCo). We assessed EF - working memory, inhibition and cognitive flexibility-, ToM and background cognitive functions - general intelligence, selective attention and speed processing - using a battery of standardized neuropsychological tests.

**Results:**

To investigate the presence of significant differences in communicative-pragmatic performance between patients and controls, we performed a 2x3 ANOVA with participant (individuals with schizophrenia, healthy control) as between-subjects factor, and the type of pragmatic phenomena (sincere, deceitful and ironic) as within-subjects factor. For each of the ABaCo subscales, we found a main effect of participant (.0001 < p. < .001), showing that experimental group performed significantly worse than control group. We also found a linear trend in pragmatic performance (.0001 < p. < .008), that revealed a linear decrease in scores depending on the pragmatic phenomenon investigated: sincere communicative acts were the easiest to understand, followed by deceit and irony. To evaluate the role of cognitive and ToM tasks on pragmatic performance in patients, we performed a regression analysis. We included relevant predictors in the model, i.e. cognitive background factors, EF and ToM. We found that the only significant predictor was ToM, that contributed to increase the quote of explained variance in the comprehension and production of linguistic sincere communicative acts (p = .005) and linguistic deceit (p. = .009).

**Discussion:**

Results showed that individuals with schizophrenia performed poorly in the comprehension and production of different kinds of pragmatic phenomena, i.e. sincere, deceitful and ironic communicative acts. This result confirms that communicative-pragmatic impairment is a core deficit in schizophrenia. In addition, we found an association between ToM and comprehension and production of sincere and deceitful communicative acts, while no association between irony and ToM was found. The results of the present investigation confirm the role that ToM can play in managing sincere and deceitful communicative acts, while do not seem to support previous evidences indicating ToM as the main factors in explaining irony understanding.

